# Synovial sarcoma of the infratemporal fossa: A case report

**DOI:** 10.3892/ol.2014.2436

**Published:** 2014-08-12

**Authors:** ALIMUJIANG WUSHOU, YA-JUN ZHAO, ZHI-MING SHAO

**Affiliations:** 1 Department of Oncology, Cancer Research Institute, Fudan University Shanghai Cancer Center, Shanghai Medical College, Fudan University, Shanghai 200032, P.R. China; 2 Department of Oral and Maxillofacial Surgery, College and Hospital of Stomatology, Xi’an Jiao Tong University, Xi’an, Shaanxi 710004, P.R. China

**Keywords:** synovial sarcoma, infratemporal fossa

## Abstract

Synovial sarcomas (SS) are high-grade soft-tissue sarcomas, predominantly found in the deep soft tissues of the lower extremities, with only 3–5% occurring in the head and neck region. Primary SS of the infratemporal fossa (ITF) is exceptionally uncommon. The present study reports the case of a 23-year-old female with an SS arising in the ITF. To the best of our knowledge, this case is only the second patient with intracranial involvement recorded in the literature. The patient was treated primarily with surgery, followed by a total of 60 Gy adjuvant radiotherapy and chemotherapy, consisting of cisplatin (25 mg/m^2^ intravenously on days one to three), epirubicin (25 mg/m^2^ intravenously on days one and two) and ifosfamide (1.8 g/m^2^ intravenously on days one to five) for three cycles. At present, two years after this multimodal therapy, the patient exhibits no signs of loco-regional recurrence or distant metastases. This study highlights the importance of a multidisciplinary approach in the diagnosis and treatment of this extremely rare entity with intracranial extension. In addition, the study reviews the English literature with regard to SS of ITF and discusses the clinicopathological features, management and outcome.

## Introduction

Synovial sarcomas (SS) are high-grade soft-tissue sarcomas that are associated with poor survival (1). The tumor cells, however, do not derive from synovial cells, but rather from cells of periarticular tissue or from mesenchymal stem cells elsewhere in the body. SS have been described at virtually every anatomical site (2). The extremities are the most common primary sites of SS. The lower extremes account for ~70% of cases (3). However, SS is uncommon in the head and neck region; 3–5% of all sarcomas arising in the head and neck are SS (4).

Due to the low clinical morbidity, concealed anatomical site, non-specific symptoms and heterogeneous histopathological features, SS in the ITF are often misdiagnosed. As a result, clinical diagnosis and treatment planning remain a challenge. To the best of our knowledge, a search of the English literature indicated that there are eight previous studies revealing nine cases of SS located in the ITF, including only one case with intracranial involvement (Table I) (5–12). There is a definitive requirement to report cases of SS in the ITF when diagnosed, as knowledge about its clinical manifestations, imaging, diagnosis, management strategies and outcome is lacking. The current study presents a case of biphasic SS located in the ITF, with intracranial involvement. The details of the clinical, radiographical, surgical and histopathological findings are reported. In addition, the reported English literature with regard to SS of ITF is systematically reviewed and the clinicopathological characteristics, treatment modality and outcome are discussed.

## Case report

**Figure 1:**
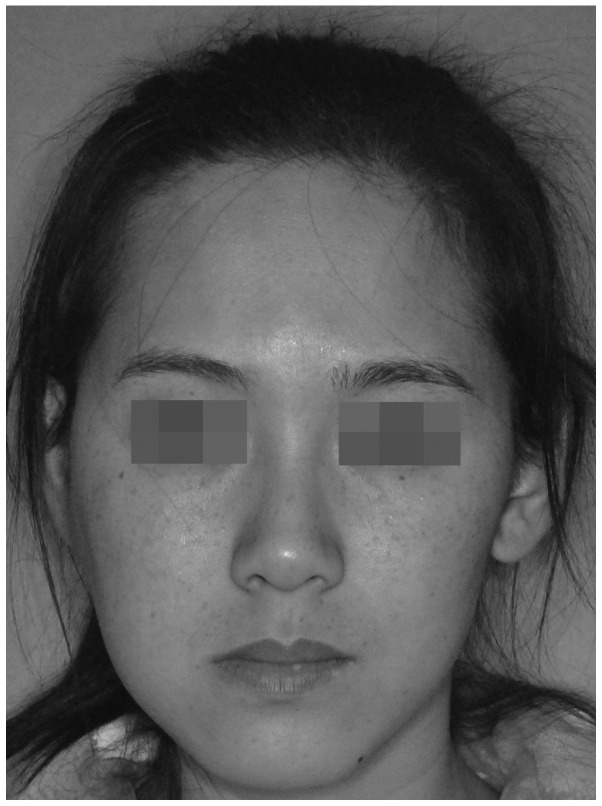
Upon physical examination, facial asymmetry and slight right-sided cheek swelling were observed.

**Figure 2:**
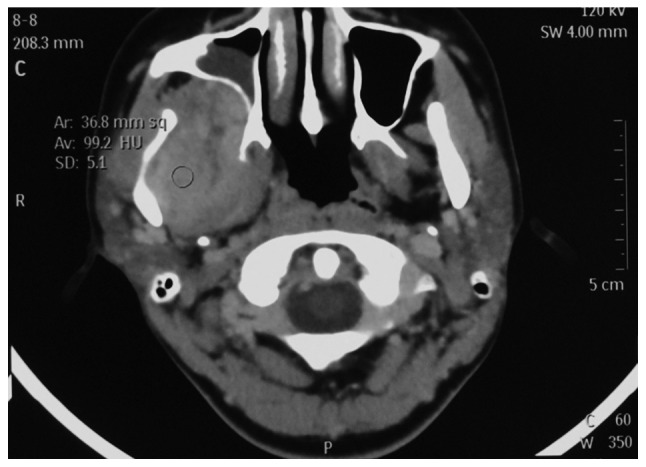
Axial computed tomography (CT) scan revealing a soft-tissue mass in the right infratemporal fossa (ITF), compressing the posterolateral wall of the right maxillary sinus and causing deformity.

**Figure 3:**
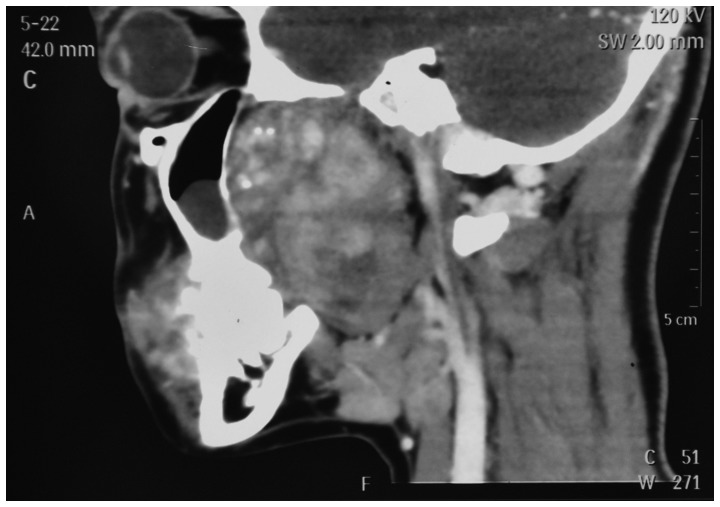
Sagittal computed tomography (CT) scan revealing a soft-tissue mass in the right infratemporal fossa (ITF) and an enlarged foramen ovale.

**Figure 4:**
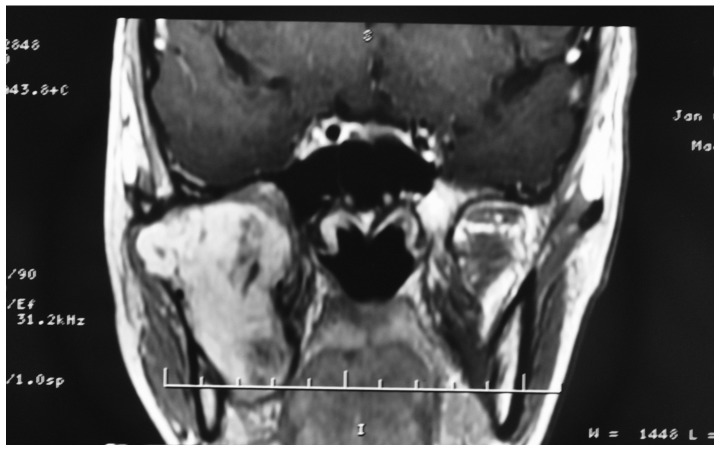
Coronal magnetic resonance imaging (MRI) scan showing a well-defined 5.1×3.6-cm mass within the right infratemporal fossa (ITF).

**Figure 5:**
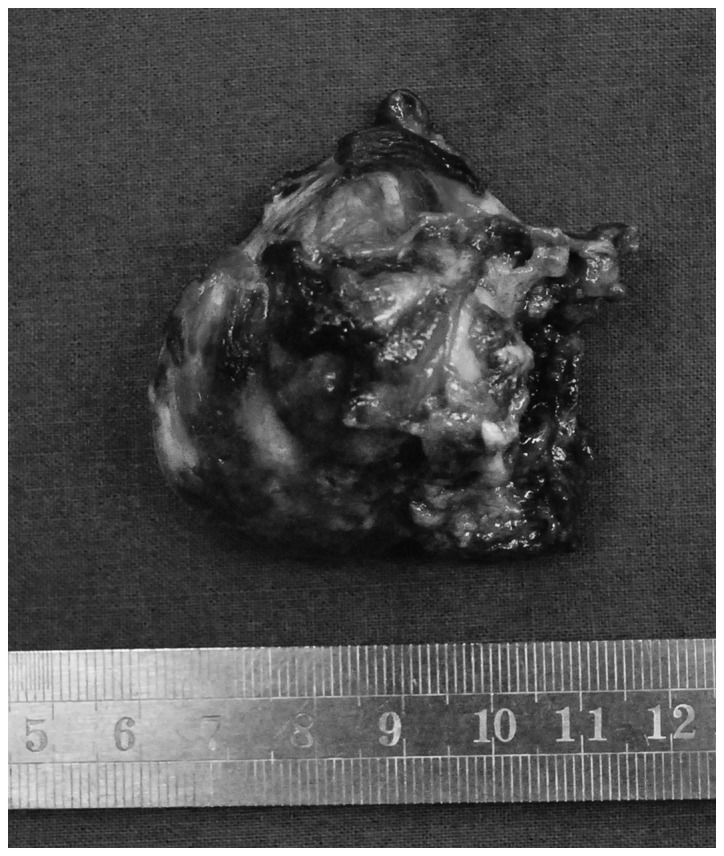
Gross identification of an ill-defined egg-shaped solid tumor, 5.1×3.6×3.2 cm in size.

**Figure 6:**
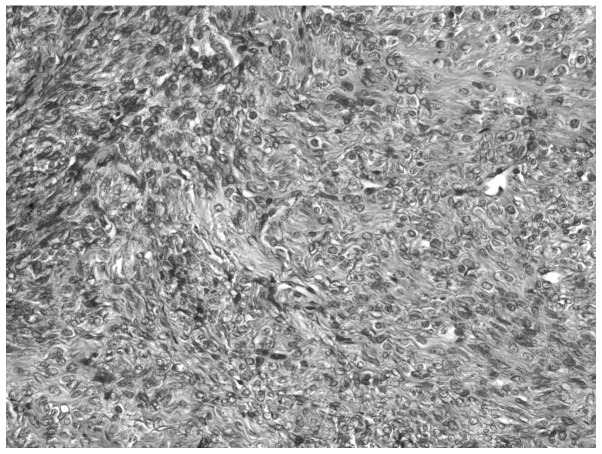
Hematoxylin and eosin-stained section composed of uniformly-shaped spindle cells, with a higher proportion of nuclei. The division of the nucleus in tumor cells is rare (original magnification ×100).

**Figure 7:**
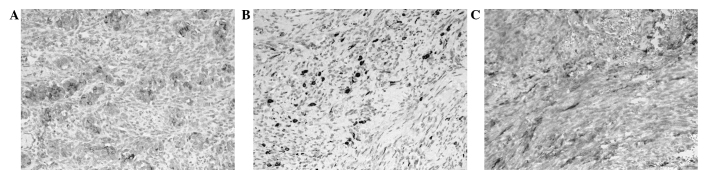
(A) Tumor cells positive for the cytokeratin (CK)7 epithelial marker. (B) Tumor cells positive for the CK19 epithelial marker. (C) Tumor cells positive for the epithelial membrane antigen (EMA) epithelial marker.

**Figure 8:**
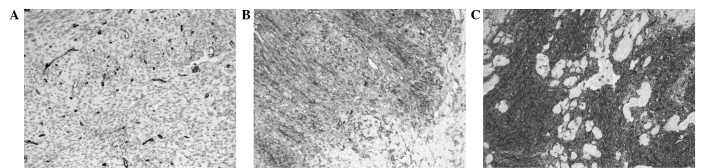
(A) The tumor cells were positive for mesenchyme marker CD34. (B) The tumor cells were positive for mesenchyme marker CD99. (C) The tumor cells were positive for mesenchyme marker Vim.

A 23-year-old female was referred to the Stomatology Hospital of Xi’an Jiao Tong University (Xi’an, China) for consultation, due to painless swelling of the right cheek that had been present for five months. The patient had no history of trauma or facial surgery. Upon physical examination, facial asymmetry and slight right-sided cheek swelling was observed (Fig. 1). The maximum extent that the mouth could be opened was 2.8 cm and the occlusal relationship was normal. A palpable mass without tenderness was present in the posterolateral wall of the right maxillary sinus; the mass was moderate in hardness and slight mobile. However, the whole body of the mass could not be assessed. There was no involvement of the oral mucosa and the lymphadenopathy was negative. The evaluation of the cranial nerves returned results within the normal limits. With the suspicion of a tumor from the right ITF, imaging examinations were performed. Computed tomography (CT) demonstrated a soft-tissue mass in the right ITF, compressing the posterolateral wall of the right maxillary sinus and causing deformity without osteolysis. The foramen ovale was also enlarged (Figs. 2 and 3). Magnetic resonance imaging (MRI) revealed a 5.1×3.6-cm mass filling the superior and inferior aspects of the ITF (Fig. 4). The patient was otherwise healthy, with complete dentition. Fine-needle aspiration cytology (FNAC) was performed pre-operatively via an intraoral approach, but it did not result in a definitive diagnosis. Based on the clinical presentation and uncommon imaging manifestations with destruction of the foramen ovale, the primary diagnosis was of a malignant tumor. Next, the patient underwent surgical excision under general anesthesia (Fig. 5). A frozen biopsy sample was obtained intraoperatively, yielding the following microscopic results: The tumor was composed of uniformly shaped spindle cells, with a higher proportion of nuclei, and plasma with a rare mitotic phenomenon (Fig. 6). Surgical margins were microscopically tumor-free and the dura was intact. There was no cerebrospinal fluid leakage. Immunohistochemistry was performed post-operatively and the results showed that the tumor cells were positive for epithelial markers, cytokeratin 7 (CK7), CK19 and epithelial membrane antigen (EMA) (Fig. 7), and mesenchymal markers, cluster of differentiation (CD)34, CD99 and Vimentin (Vim) (Fig. 8). The tumor cells were negative for p63, smooth muscle actin and S-100. The tumor was subsequently diagnosed as biphasic SS. Treatment with a total of 60 Gy adjuvant radiotherapy and chemotherapy, consisting of cisplatin (25 mg/m^2^ intravenously on days one to three), epirubicin (25 mg/m^2^ intravenously on days one and two) and ifosfamide (1.8 g/m^2^ intravenously on days one to five) for three cycles was administered to prevent local recurrence and distant metastasis. At the 24-month follow-up neither local recurrence nor metastatic disease were apparent. Written informed patient consent was obtained for publication of this study.

## Discussion

A review of the English literature revealed that a total of 10 cases, including the present case (Table I), regarding SS arising from the ITF have been reported, making this an extremely rare entity. This poses a challenge for physicians to define its clinical behavior and to standardize a management strategy. All reported series of SS in the head and neck are sporadic and comparisons are difficult. According to the largest series from the MD Anderson Cancer Center (Houston, TX, USA), the median age of patients with SS of the head and neck was 29 years (mean, 30.6 years; range, 5–55 years), and 73% of occurrences were male and 27% were female (4). Another study also reported similar age ranges (13). Of the ten cases identified in the present review, the ages ranged between 7 and 82 years, with a mean of 38.6 years. In contrast to the MD Anderson Cancer Center study, there was a female predominance, with a female to male ratio of 4:1.

The tumor site determines the clinical presentation of the head and neck SS (4). The ITF, by virtue of its relatively concealed location, is inaccessible for clinical examination of the tumor in the early stages. Space-occupying lesions in this area may continue to grow unnoticed for a considerable period. Clinically, SS of the ITF appears as a deep-seated, painless and slow-growing mass, and is usually asymptomatic until it attains a size sufficient enough to create pressure on the adjacent structures. In the current review, the mean tumor size was 5.9 cm (range, 3.2–13 cm), and it took 5–12 months for these patients to seek first medical care. In the cases with reported symptoms, SS in the ITF manifested with painless check swelling (1/7; present case), local pain (4/7), restriction of mouth opening (2/7) and migraines (1/7). In case 4, the tumor presented with local pain associated with asthenia, anorexia and weight loss (12.0 kg) over 6 months of progression. In cases 3 and 4, tumors in the advanced stage invaded the oral cavity and presented with oral masses; thus, the growth was similar in appearance to squamous cell carcinoma arising from the maxillary sinus (particularly from the posterior wall) and retromolar triangle (Table I).

Tumors of the ITF present with a wide spectrum of pathologies, both benign and malignant (14). A smaller number of tumors, particularly those rare entities such as SS, originate from the tissues in this space, making them difficult to diagnose correctly. SS of the head and neck may usually mimic the benign neoplasms in CT and MRI, with well-defined, smooth margins and a lack of aggressive infiltration (11,15). Indeed, its heterogeneity in appearance with septations, hemorrhage, cysts, calcification or multilocularity should raise the suspicion of an SS (15). CT and/or MRI was performed in 9 cases of SS in association with the ITF, including the present case. Three cases presented with a heterogeneous mass and calcification (3/9); one case with septation (1/9); another case presented with a homogeneous cystic mass (1/9); seven with tumors extending into the surrounding soft tissue (7/9); six presented with infiltration into the bony structures except the skull base (6/9); and two cases exhibited intracranial extension (foramen ovale) (2/9). As indicated by the present review, physical examinations and CT/MRI imaging were extremely useful to disclose local invasion and metastasis at the time of presentation. Lymphadenopathy was not detected in any of these cases.

The diagnosis of SS is made on the basis of its relatively distinctive, yet markedly variable, histopathological appearance, in conjunction with histochemical findings, immunohistochemistry, electron microscopy and cytogenetic analysis, which have proved valuable in confirming the morphologic diagnosis (16,17). Two morphologically distinct, but histogenetically-related cell types form SS and cause the characteristic biphasic pattern. SS form a continuous histopathological spectrum, with biphasic, monophasic epithelial, monophasic fibrous and poorly-differentiated (round cell) types, depending on the relative prominence of the two cell populations and the degree of differentiation (1). Biphasic SS is effectively diagnosed by its unique histopathological features, however, it is difficult to diagnose monophasic SS. Therefore, immunohistochemistry has a significant role in the diagnosis of SS. The present case analysis demonstrated a monophasic:biphasic ratio of 1:1. The literature review demonstrated that an immunohistochemical analysis was performed in seven of the known cases, including four cases of biphasic SS, two cases of monophasic SS and another unspecified case. The most commonly positive epithelial markers were EMA (6/7) and CK (5/7), while the mesenchymal markers were CD99 (4/7) and Vim (4/7). There was no significant difference between monophasic and biphasic SS with respect to their immunohistochemical features. The differential diagnosis for this condition includes fibrosarcoma, Ewing’s sarcoma, leiomyosarcoma, malignant nerve sheath tumors, hemangiopericytoma and squamous cell carcinoma (18).

In the present case, the diagnosis of biphasic SS was made from the histopathological findings and supporting immunohistochemical features. FNAC was performed pre-operatively and yielded no definitive diagnosis. In fact, of the four patients that underwent FNAC in the reviewed cases, only one case led to a confirmed diagnosis, indicating the limited nature of this technique as a routine diagnostic procedure.

The optimal approach to the treatment of this malignancy remains undefined and there is no standard treatment protocol for SS of the head and neck (4). From the analysis of the nine previous reports, it was apparent that the treatment protocol of the SS arising from the ITF was inconsistent, consisting of the following combinations: Surgery only (2/6), surgery and radiotherapy (1/6), surgery and chemotherapy (1/6), surgery, chemotherapy and radiotherapy (3/6), and radiotherapy and chemotherapy (1/6). Generally, radical surgery represented the first approach (6/7). However, a radical excision with wide margins is rarely possible due to the anatomical site. With respect to the present patient with intracranial extension, surgery and chemoradiotherapy were applied.

For soft-tissue sarcomas in general, the prognosis is associated with the resection margins (19). SS, one of the highly malignant tissue sarcomas, remains a disease with a poor prognosis, having an overall five-year survival rate of 57% (18). For head and neck SS, the 5-year disease-specific survival rate has been recorded as 72%, and survival rates have been found to be associated with tumor location, size, and extension (4). Of the eight patients with a known outcome in the literature, the median follow-up period was 71.5 months (range, 12–192 months). As a result, six patients with combined therapy were disease-free (6/8). One case thatwas treated exclusively with chemotherapy and radiotherapy, but not surgery, remained unchanged following 180 months of follow-up, with no signs of tumor aggressiveness. One patient developed multiple pulmonary and pleural metastases, and eventually succumbed to the disease 14 months post-operatively. It is noteworthy that this patient received only surgery, and exhibited a large tumor of 7.0 cm in diameter, plus surrounding soft tissue extension and bony infiltration. Another patient with recurrent disease was a 7-year-old female who suffered from multiple lung and chest wall metastases at 192 months post-treatment. However, further information concerning the clinicopathological characteristics and treatment options in this case are not available. These cases may advocate the importance of multidisciplinary management in this rare entity with or without surrounding soft/bony tissue extension.

Recently, Aslan *et al* reported a similar case of SS in the ITF with intracranial extension (5). In this case, the tumor invaded the foramen ovale, but was not involved with the surrounding soft tissue or bony structures; the mass was surgically removed en-bloc and received chemoradiotherapy post-operatively. Unlike the present case, the tumor was small at only 3.3 cm in diameter, but was much more aggressive; it destroyed a 0.5×0.5-cm area of bone posterolateral to the foramen ovale, although the dura remained intact. Surgicel (Johnson & Johnson Medical Ltd., Zug, Switzerland) was applied to the destroyed area and there was no cerebrospinal fluid leakage. Following the use of multidisciplinary management, the patient had a good prognosis at the 12-month radiological follow-up.

Due to the limited number of ITF cases, every new case will highlight novel information about management strategies and prognosis. The current study presented an extremely rare case of primary SS in the ITF with intracranial involvement. Albeit with only a short follow-up period, the patient achieved good outcome after multimodal therapy. Based on a review of the literature, SS in the ITF is insidious due to its special anatomical features in the skull base, and it may not be noticed until there is impairment of function and the appearance of symptoms. CT and MRI are useful non-invasive diagnostic tools, and the final diagnosis of SS is made on the basis of unique pathological and immunohistochemical findings. In this rare entity with or without surrounding soft/bony tissue extension, multimodal therapy with surgical excision followed by early post-operative chemoradiotherapy can be a promising factor controlling local recurrence and distant metastasis.

## Figures and Tables

**Table I:** Cases of SS of the ITF reported in the English literature.

Cases	1	2	3	4	5	6	7	8	9	10
Age, years	23	30	26	72	72	31	46	61	18	7
Gender	Female	Male	Female	Female	Male	Female	Female	Female	Female	Female
Case history, months	5	12	Unspecified	6	Unspecified	6	Unspecified	Unspecified	Unspecified	Unspecified
Symptoms	Swelling	LP	RMO	LP	Unspecified	RMO, LP	Migraines	LP	Unspecified	Unspecified
Radiology	CT, MRI	CT, MRI	CT	CT, MRI	MRI	MRI	CT, MRI	CT	MRI	Unspecified
Tumor diameter, cm	5.1	3.3	3.2	7.0	13.0	5.0	4.7	Unspecified	Unspecified	Unspecified
TMM	Heterogeneous, septation	Heterogeneous, calcifications	Homogeneous	Heterogeneous, necrosis, calcification	Homogeneous cystic mass	Heterogeneous	Heterogeneous	Heterogeneous, calcifications	Heterogeneous	Unspecified
SSTE	No	No	Yes	Yes	Yes	Yes	Yes	Yes	Yes	Unspecified
Bony infiltration	Yes	No	Yes	Yes	No	No	Yes	Yes	Yes	Unspecified
ICE	Yes	Yes	No	No	No	No	No	No	No	Unspecified
Lymphadenopathy	Negative	Negative	Negative	Negative	Unspecified	Negative	Negative	Unspecified	Unspecified	Unspecified
Preoperetive biopsy	Yes (FNC)	No	Yes (FNC, IB)	Yes (FNC, IB)	Unspecified	Unspecified	Yes (FNC, IB)	Unspecified	Unspecified	Unspecified
DB-FNAC	No	No	Yes	No	Unspecified	Unspecified	No	Unspecified	Unspecified	Unspecified
Surgery type	En-bloc	En-bloc	En-bloc	En-bloc	Unspecified	En-bloc	En-bloc	Unspecified	None	Unspecified
IFB	Yes	Unspecified	Yes	Yes	Unspecified	Unspecified	Yes	Unspecified	None	Unspecified
Margin status	Negative	Negative	Negative	Negative	Unspecified	Negative	Negative	Unspecified	Unspecified	Unspecified
PD	Biphasic	Biphasic	Biphasic	Biphasic	Unspecified	Monophasic	Monophasic	Monophasic	Monophasic	Unspecified
ADT	Yes (IHC)	Yes (IHC)	Yes (IHC)	Yes (IHC)	Unspecified	Yes (IHC)	Yes (IHC)	Unspecified	Unspecified	Unspecified
IHC positive for	EMA, Vim CD99, CK7, CK19, CD34	EMA, CK	EMA, Vim, CK, Cal, Bcl-2, S-100	Vim, CD99	Unspecified	EMA, Vim, CK, CD99	EMA, CK, Cal, Bcl-2, S-100	Unspecified	Unspecified	EMA, CK, CD99
Treatment	S+C+R	S+C+R	S+C	S	Unspecified	S	S+C+R	S+R	C+R	Unspecified
PMW	Yes (PET-CT)	Yes (MRI)	Yes	Yes	Unspecified	Unspecified	Yes	Unspecified	Yes	Unspecified
Follow-up (m)	24	12	42	14	Unspecified	Unspecified	12	96	180	192
Outcome	NED	NED	NED	MPPM	Unspecified	Unspecified	NED	NED	NED	MLCM
Survival status	Alive	Alive	Alive	Death	Unspecified	Unspecified	Alive	Alive	Alive	Unspecified
Year	2013	2012	2012	2010	2008	2008	2007	2001	2001	2000
Country	China	Turkey	India	Spain	Canada	China	USA	France	France	USA
First author	Present case	Aslan *et al*	Dhawan *et al*	Conejeros *et al*	O’Sullivan *et al*	Wang *et al*	Lai *et al*	Rangheard *et al*	Rangheard *et al*	Silverman *et al*
Reference		(5)	(6)	(7)	(8)	(9)	(10)	(11)	(11)	(12)

SS, synovial sarcoma; ITF, infratemporal fossa; LP, local pain; RMO, restriction of mouth opening; CT, computed tomography; MRI, magnatic resonance imaging; TMM, typical imaging manifestations; SSTE, surrounding soft tissue extension; ICE, intracranial extension; DB, diagnosed by; FNAC, fine-needle aspiration cytology; IB, incisional biopsy; IFB, intraoperative frozen biopsy; PD, pathological diagnosis; ADT, ancillary diagnostic techniques; IHC, immunohistochemistry; S, surgery; C, chemotherapy; R, radiotherapy; PMW, post-operative metastatic workup; PET-CT, positron emission tomography; NED, no evidence of disease; MPPM, multiple pulmonary and pleural metastases; MLCM, multiple lung and chest wall metastases; Vim, vimentin; EMA, epithelial membrane antigen; CD, cluster of differentiation; CK, cytokeratin; Cal, calponin; Bcl, B-cell lyphoma.
